# Regional references vs. international standards for assessing weight and length by gestational age in Lithuanian neonates

**DOI:** 10.3389/fped.2023.1173685

**Published:** 2023-06-14

**Authors:** Ruta Morkuniene, Tim J. Cole, Egle Marija Jakimaviciene, Agne Bankauskiene, Jelena Isakova, Nijole Drazdiene, Vytautas Basys, Janina Tutkuviene

**Affiliations:** ^1^ Department of Anatomy, Histology and Anthropology, Institute of Biomedical Sciences, Faculty of Medicine, Vilnius University, Vilnius, Lithuania; ^2^ UCL Great Ormond Street Institute of Child Health, London, United Kingdom; ^3^ Department of Human and Medical Genetics, Institute of Biomedical Sciences, Faculty of Medicine, Vilnius University, Vilnius, Lithuania; ^4^ Health Information Center, Institute of Hygiene, Vilnius, Lithuania; ^5^ Clinic of Children’s Diseases, Institute of Clinical Medicine, Faculty of Medicine, Vilnius University, Vilnius, Lithuania; ^6^ Division of Biological, Medical and Geosciences, Lithuanian Academy of Sciences, Vilnius, Lithuania

**Keywords:** birth, growth charts, newborns, preterm (birth), SGA (small for gestational age), LGA (large for gestational age), global, regional

## Abstract

**Introduction:**

There is no global consensus as to which standards are the most appropriate for the assessment of birth weight and length. The study aimed to compare the applicability of regional and global standards to the Lithuanian newborn population by sex and gestational age, based on the prevalence of small or large for gestational age (SGA/LGA).

**Materials and Methods:**

Analysis was performed on neonatal length and weight data obtained from the Lithuanian Medical Birth Register from 1995 to 2015 (618,235 newborns of 24–42 gestational weeks). Their distributions by gestation and sex were estimated using generalized additive models for location, scale, and shape (GAMLSS), and the results were compared with the INTERGROWTH-21st (IG-21) standard to evaluate the prevalence of SGA/LGA (10th/90th centile) at different gestational ages.

**Results:**

The difference in median length at term between the local reference and IG-21 was 3 cm–4 cm, while median weight at term differed by 200 g. The Lithuanian median weight at term was higher than in IG-21 by a full centile channel width, while the median length at term was higher by two channel widths. Based on the regional reference, the prevalence rates of SGA/LGA were 9.7%/10.1% for boys and 10.1%/9.9% for girls, close to the nominal 10%. Conversely, based on IG-21, the prevalence of SGA in boys/girls was less than half (4.1%/4.4%), while the prevalence of LGA was double (20.7%/19.1%).

**Discussion:**

Regional population-based neonatal references represent Lithuanian neonatal weight and length much more accurately than the global standard IG-21 which provides the prevalence rates for SGA/LGA that differ from the true values by a factor of two.

## Introduction

The health status of a newborn is reflected by its body size at birth. Therefore, the assessment of birth weight and length by applying appropriate standards not only plays an essential role in good clinical care but is also sustainable with an increasing evidence of preventable adverse growth-related outcomes (1).

When considering neonatal body size, two groups with an increased risk for negative perinatal outcomes and future cardio-metabolic changes have been identified (2): infants born Small for Gestational Age (SGA) and infants born Large for Gestational Age (LGA). The WHO expert committee defined SGA as infants born below the 10th centile of birth weight for gestational age and sex specific reference population (3). Accordingly, LGA is defined as birth weight above the 90th centile. Importantly, SGA refers to both, infants who are constitutionally small and fall within the lower tail of the distribution and those with intra-uterine growth restriction (IUGR) due to various adverse factors (4).

Moreover, a range of recent publications established the relationship between the preterm newborn body's parameters at birth, postnatal growth restriction and later growth failure with short- and long-term health consequences (5–7).

Thus, from a clinical perspective, the priority would be to reduce health effects related to size at birth by providing a preventive and promotive care, and monitoring for complications (8). Adequate early nutrition is extremely important in clinical practice to foster optimal neurodevelopmental outcomes to avoid negative consequences of aggressive nutritional approaches in the future. However, to provide the mentioned nutrition, doctors must properly evaluate the newborn size according to their gestational age (small, large, and appropriate) at birth. This classification is difficult to achieve, especially for preterm infants who are a unique and highly heterogeneous group. Currently, neonatal standards and references are the most widely used tools for categorizing newborns into sub-groups.

At present, several types of charts for growth assessment of premature newborns are available. One of the commonly used types is intrauterine growth charts based on cross-sectional anthropometric measurements taken from infants of varying gestational ages at birth presented as the gold standard for premature infant growth (9). However, the application of intrauterine growth rates to VLBW infants in an extra-uterine environment is considered controversial (10, 11). Instead, a separate set of postnatal growth curves is promoted. This group of charts reflects the longitudinal growth of preterm infants experiencing various clinical conditions which might be treated with different nutritional management plans (12). And while postnatal growth curves increase our understanding of postnatal preterm born child growth, they also have their drawbacks, such as being too highly influenced by medical and nutritional support practices applied to the sample size (13).

Another point of debate is the choice between customized and population-based references. Proponents of customized charts claim that they improve the prediction of adverse perinatal outcomes and differentiate better between “small-but-healthy” infants and growth-restricted ones (14, 15). Opponents argue that the existing customization models may be confusing pathological and physiological causes of “smallness” and that customizing birthweight centiles for maternal characteristics has little justification (16, 17). Instead, the best estimate of a given infant's birthweight is claimed to be the one that is close to the population average (16).

A similar discussion arises when choosing between the regional or global references (9, 18–21). Recently, the Intergrowth-21st (IG-21) consortium proposed to adopt their single set of international standards for assessment of newborn size and physical status but found little support (22–24). With the rising evidence that there is a normal physiological variation between different countries and ethnic groups, the “one-size-fits-all” standard seems to fit less than expected.

Considering all the above, in this study, we have set several objectives: (a) to construct the national birth weight/length reference values and curves for Lithuanian newborns from 22 to 42 GA weeks; (b) to compare the results with the recently adopted global IG-21 (18, 20) standards; (c) to assess the diagnostic accuracy of clinical practice in identifying and classifying newborns of various gestational age as SGA and LGA infants, by using different tools.

## Materials and methods

### Study design and sample selection

**Figure 1:**
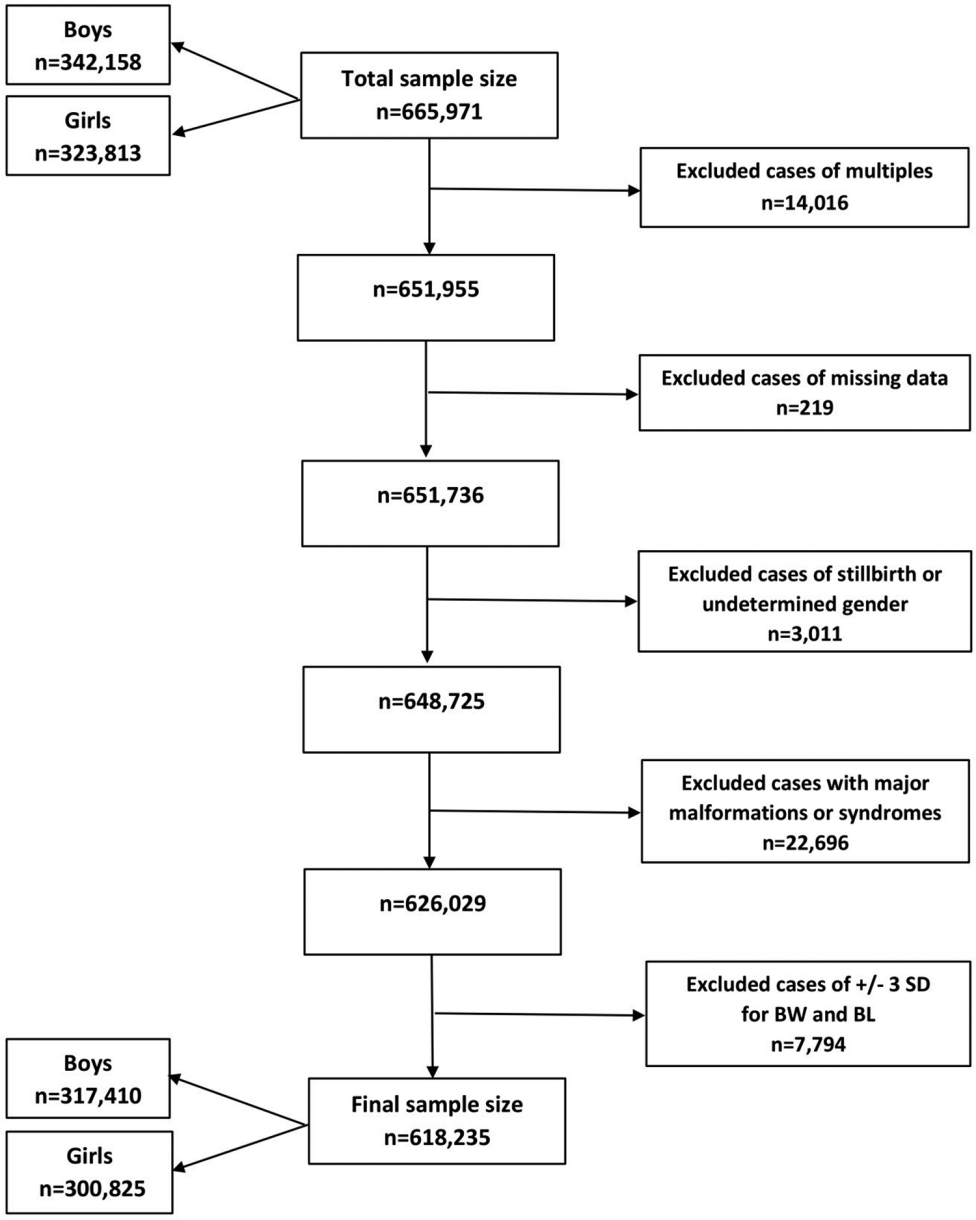
Flow diagram of the sampling procedure and exclusion criteria in this study. BW, birth weight; BL, birth length; *n*, number; SD, standard deviation.

Birth data from the Lithuanian Medical Data of Births for the period of 1995–2015 were retrieved from the Health Information Center of the Institute of Hygiene in Vilnius, Lithuania, including liveborn singletons of between 22 and 42 completed weeks of gestational age (GA). We excluded multiple births, stillbirths, infants with undetermined sex or major congenital malformations and syndromes, and those with incomplete data. Also, the cases where weight or length were more than 3 standard deviations (SD) from the mean, following WHO standards (25), were excluded. In total, 618,235 newborns were included. Figure 1 shows the flow diagram.

### LMST method

The LMS method estimates growth reference centiles by modelling any skewness present in the measurement distribution (26). It assumes that a power transformation can normalize the data by adjusting for skewness. LMST method (27) is an extension of the LMS method that models both skewness and leptokurtosis using the Box-Cox *t* (BCT) distribution. It can be used to model excess kurtosis over the normal distribution (leptokurtic data) when the Box-Cox transformation fails to transform the data close to normality due to the presence of kurtosis. The BCT distribution is defined by a power transformation, *Y^ν^*, which follows a truncated *t* distribution that is shifted and scaled. The truncated *t* distribution has a degrees of freedom parameter, *τ*. The BCT distribution is characterized by four parameters and is denoted as BCT (*µ, σ, ν, τ*). The parameters *µ*, *σ*, *ν* and *τ* may be interpreted as relating to location (median), scale (centile-based coefficient of variation), skewness (power transformation to symmetry) and kurtosis (degrees of freedom), respectively.

### Statistical analysis

Generalized additive models for location, scale, and shape (GAMLSS) were fitted to obtain the centile (3rd, 10th, 25th, 50th, 75th, 90th and 97th) reference values and curves of birth weight/length by GA and sex separately (26). The LMST method (BCT distribution) was applied to the data obtained on each sex and each measurement. The resulting main centiles (3rd, 10th, 50th, 90th and 97th) from 24 gestational weeks were compared with their counterparts in IG-21 (18, 20). The analysis was carried out using the GAMLSS package (version 4.3-3) of R 4.0.3 (28).

Two published standards of the IG-21 project (18, 20) were presented for both sexes for every gestational week and day separately (e.g., 30 + 0, 30 + 1), while GA of the present study was recorded as complete gestational weeks (e.g., 30, 31). Therefore, the comparison of present study with IG-21 project by GA was made comparing the middle day of gestational week of IG-21 (i.e., 30 + 3) which referred to the mean of birth weight/length of the certain gestational week. The validity of this procedure was checked and proved mathematically. The frequency and percentage of neonates meeting the criteria for SGA defined as birth weight below the 10th centile and LGA defined as birth weight above the 90th centile were determined and compared using the cut-off values based on our reference and the IG-21.

## Results

The sample size of our study (Table 1) increased steeply with gestational age from less than 50 neonates at 22–24 gestational weeks to more than 140,000 at term for each sex. The mean birth weight of boys was ∼50–100 g larger than for girls at preterm gestations, and larger by ∼100–180 g at term and post-term periods. The difference in mean birth length between sexes varied mainly from 0.1 to 0.6 cm at preterm period, and from 0.6 to 0.8 cm at later gestations (Table 1).

**Table 1:** Birth weight (g) and length (cm) of Lithuanian newborns by sex and gestational age (GA).

Boys (*n* = 317,410)					GA (in weeks)	Girls (*n* = 300,825)				
Count (*n*)	Weight (g)		Length (cm)			Count (*n*)	Weight (g)		Length (cm)	
	Mean	SD	Mean	SD			Mean	SD	Mean	SD
1*	915.0	–	37.0	–	**22**	8	635.3	111.0	29.0	3.2
12	706.9	115.0	31.8	2.2	**23**	13	649.9	83.2	30.9	1.9
38	773.0	109.0	33.2	3.0	**24**	46	715.0	96.3	33.0	2.6
93	849.3	121.6	33.7	2.8	**25**	82	798.0	105.5	33.6	2.2
136	966.9	144.7	35.4	2.4	**26**	139	892.4	157.3	34.7	2.8
196	1,106.9	181.0	37.0	2.8	**27**	174	1,028.6	187.9	36.3	3.3
304	1,229.3	193.9	38.1	2.9	**28**	239	1,181.3	237.4	37.9	3.0
291	1,375.5	235.2	39.8	2.9	**29**	255	1,314.2	236.0	39.2	3.1
445	1,542.7	243.4	41.4	2.8	**30**	405	1,485.8	253.2	40.9	3.1
488	1,756.5	263.3	42.9	2.9	**31**	431	1,662.7	298.7	42.3	3.0
849	1,946.9	288.2	44.3	2.6	**32**	720	1,886.5	309.4	44.0	2.8
1,116	2,152.2	326.1	45.6	2.6	**33**	909	2,061.3	325.4	45.0	2.7
1,877	2,381.1	351.6	46.8	2.5	**34**	1,609	2,289.8	355.2	46.3	2.5
2,858	2,585.0	354.1	47.8	2.3	**35**	2,421	2,482.8	355.9	47.4	2.3
5,592	2,772.3	368.8	48.9	2.3	**36**	4,967	2,679.1	360.8	48.5	2.2
13,141	3,124.5	404.4	50.7	2.2	**37**	11,219	3,009.5	393.0	50.1	2.1
33,850	3,363.7	417.5	51.7	2.1	**38**	29,873	3,224.0	407.3	51.1	2.1
68,016	3,540.8	413.1	52.5	2.1	**39**	63,919	3,397.1	405.0	51.8	2.1
144,625	3,670.0	421.4	53.0	2.1	**40**	141,902	3,519.9	405.9	52.3	2.1
40,947	3,773.0	425.0	53.5	2.2	**41**	39,209	3,609.1	415.1	52.7	2.1
2,535	3,774.2	492.0	53.4	2.4	**42**	2,285	3,594.3	449.3	52.6	2.3

*n*, count; Mean, mean; SD, standard deviation.

* An actual case that does not exceed the critical deviation from birth weight/length.

**Figure 2:**
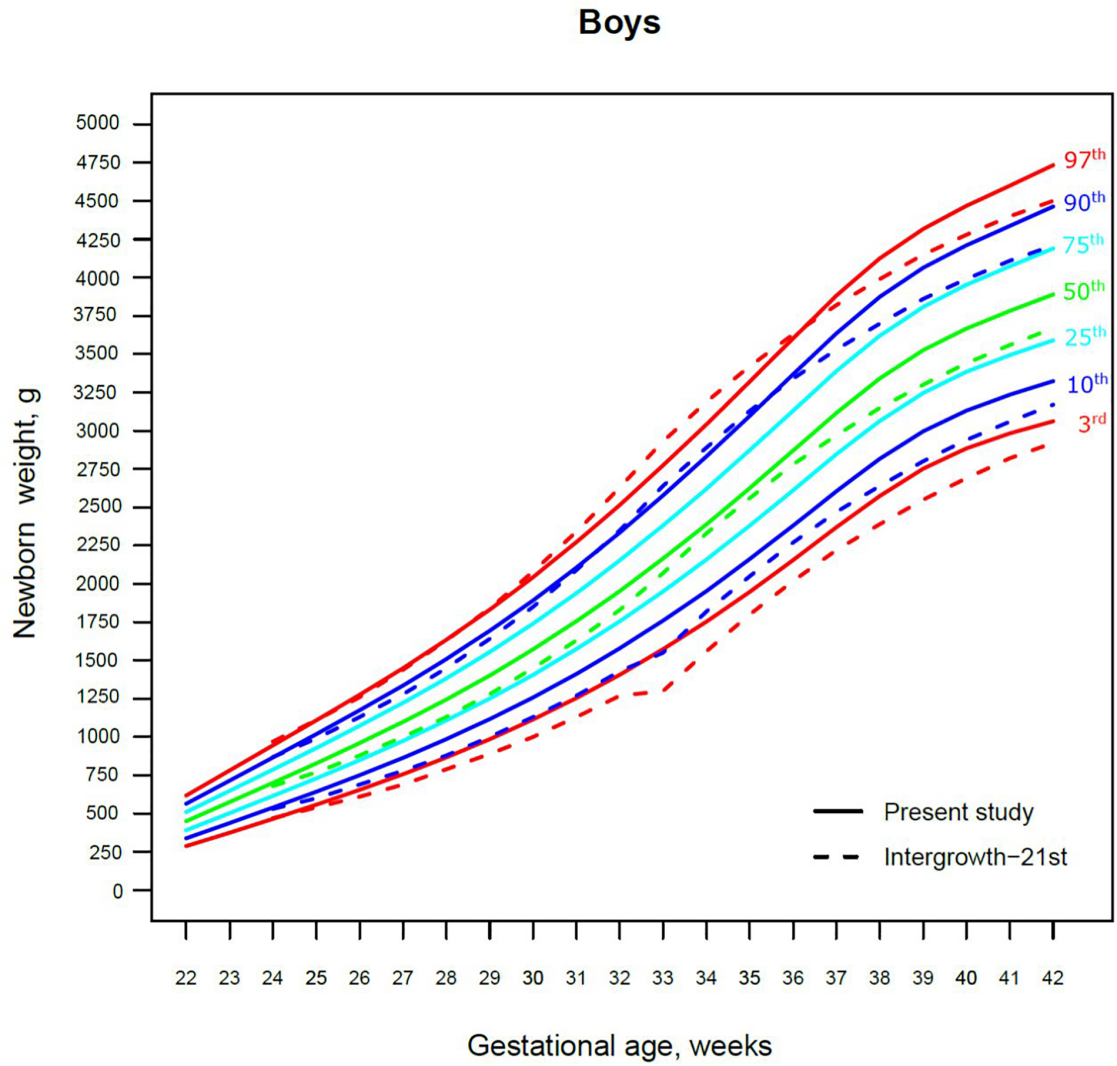
The 3rd, 10th, 25th, 50th, 75th, 90th and 97th smoothed centile curves for birth weight (g) in Lithuanian boys and the 3rd, 10th, 50th, 90th and 97th centiles for INTERGROWTH-21st.

**Figure 3:**
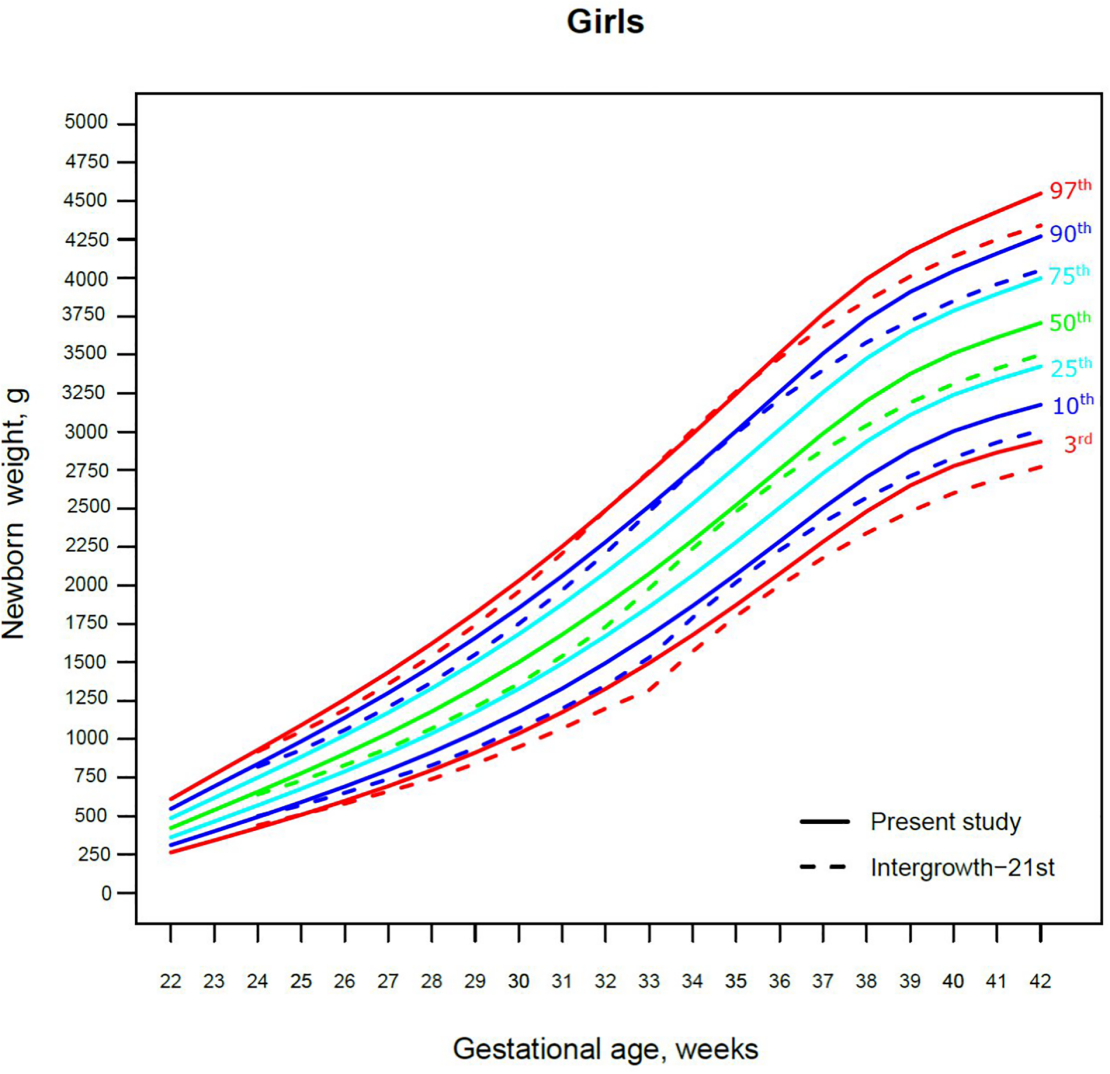
The 3rd, 10th, 25th, 50th, 75th, 90th and 97th smoothed centile curves for birth weight (g) in Lithuanian girls and the 3rd, 10th, 50th, 90th and 97th centiles for INTERGROWTH-21st.

**Figure 4:**
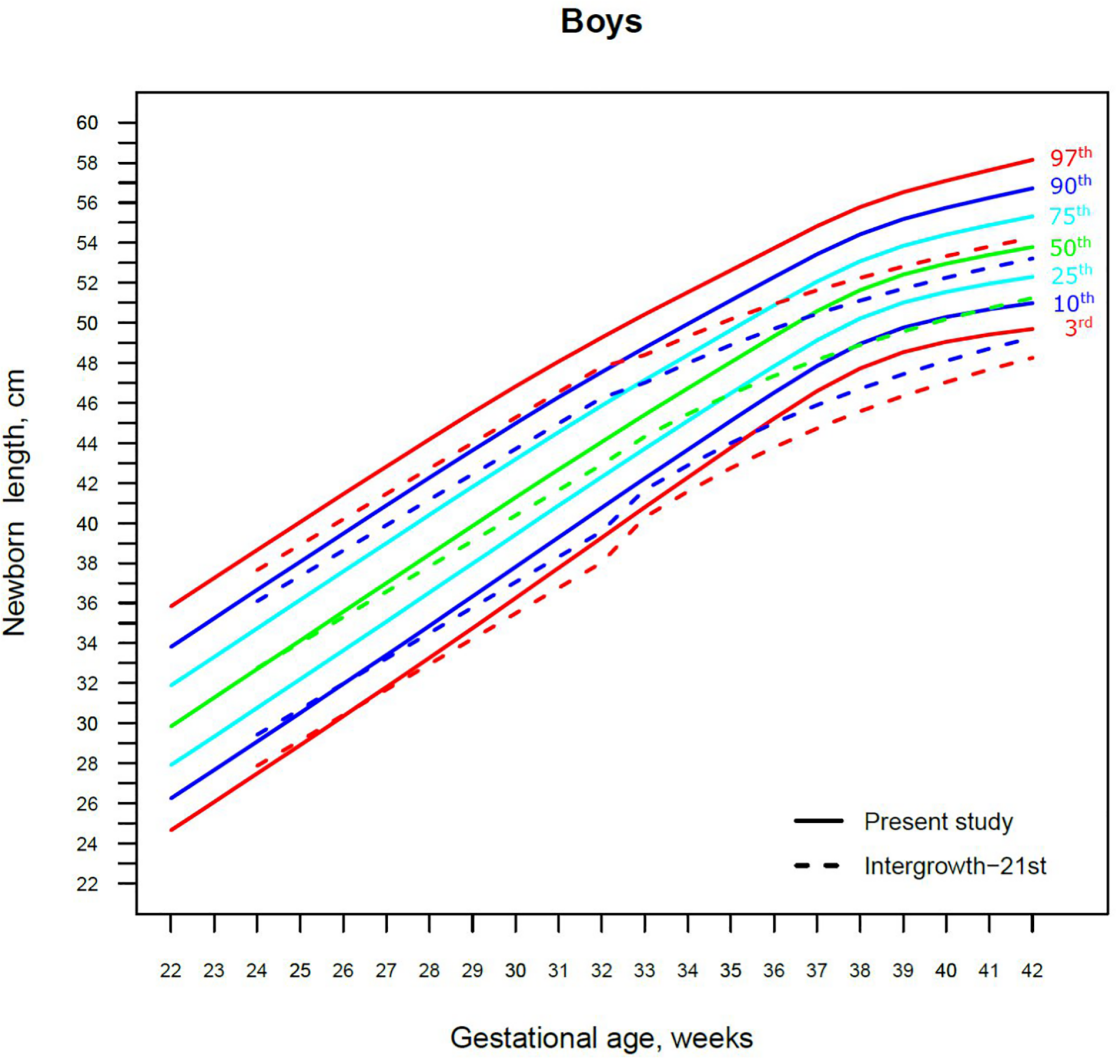
The 3rd, 10th, 25th, 50th, 75th, 90th and 97th smoothed centile curves for birth length (cm) in Lithuanian boys and the 3rd, 10th, 50th, 90th and 97th centiles for INTERGROWTH-21st.

**Figure 5:**
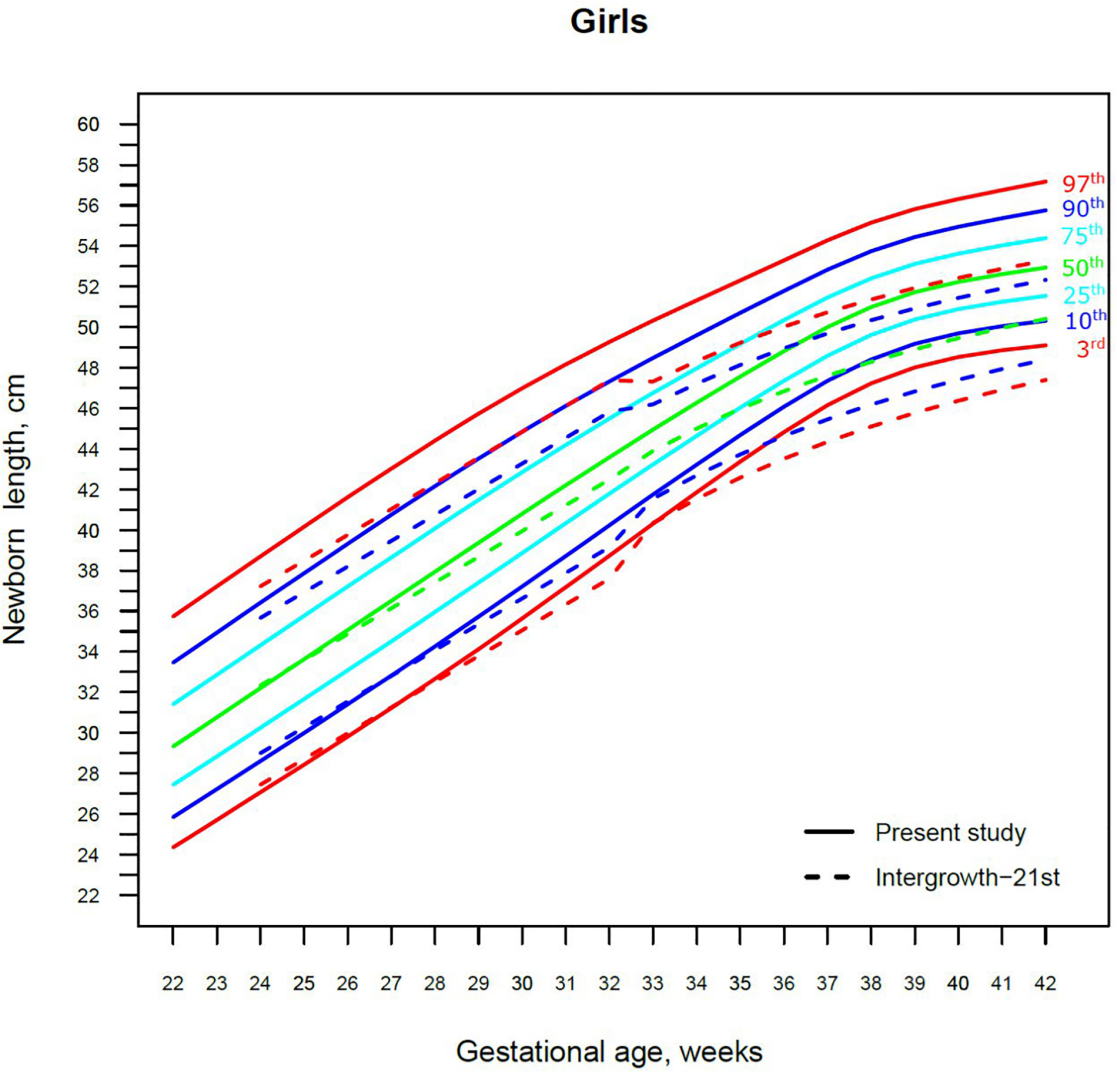
The 3rd, 10th, 25th, 50th, 75th, 90th and 97th smoothed centile curves for birth length (cm) in Lithuanian girls and the 3rd, 10th, 50th, 90th and 97th centiles for INTERGROWTH-21st.

Our study constructed the 3rd, 10th, 25th, 50th, 75th, 90th, and 97th smoothed centile curves according to gestational age and sex for birth weight and length of Lithuanian newborns (Figures 2–5). The variability of birth weight rose with gestational age for both sexes. In contrast, the variability of birth length fell with increasing gestation, and negative skewness in the distribution was evident (Figures 2–5). Tables 2, 3 demonstrate the values of smoothed gestational age- and sex-adjusted centiles (3rd–97th) for birth weight/length of Lithuanian newborns. The LMS parameters for birth weight/length by sex and gestational age are presented in Supplementary Tables S1, S2.

**Table 2:** Smoothed centiles for Lithuanian birth weight (g) by sex and gestational age (GA).

Boys							GA (in weeks)	Girls						
Birth weight centiles								Birth weight centiles						
3	10	25	50	75	90	97		3	10	25	50	75	90	97
288	339	391	450	510	564	618	**22**	264	312	363	422	486	546	610
375	438	503	575	649	716	783	**23**	342	402	465	539	618	693	770
465	539	615	701	788	867	946	**24**	424	494	569	657	750	838	930
558	643	730	829	928	1,019	1,109	**25**	509	590	677	778	885	987	1,092
655	751	850	960	1,073	1,174	1,276	**26**	598	691	789	904	1,025	1,140	1,259
758	865	975	1,098	1,223	1,337	1,450	**27**	694	798	909	1,038	1,173	1,302	1,435
867	986	1,108	1,245	1,383	1,509	1,634	**28**	798	914	1,038	1,181	1,331	1,474	1,622
985	1,117	1,251	1,402	1,555	1,694	1,832	**29**	912	1,041	1,177	1,335	1,501	1,657	1,820
1,114	1,258	1,406	1,572	1,740	1,892	2,044	**30**	1,037	1,179	1,328	1,501	1,682	1,854	2,031
1,254	1,412	1,574	1,756	1,939	2,106	2,272	**31**	1,176	1,330	1,492	1,680	1,877	2,062	2,254
1,407	1,579	1,755	1,953	2,153	2,335	2,515	**32**	1,328	1,495	1,670	1,872	2,084	2,283	2,489
1,573	1,759	1,950	2,165	2,381	2,578	2,773	**33**	1,496	1,674	1,861	2,077	2,303	2,515	2,733
1,753	1,954	2,159	2,389	2,621	2,832	3,042	**34**	1,677	1,867	2,066	2,294	2,532	2,756	2,986
1,948	2,162	2,380	2,625	2,873	3,097	3,320	**35**	1,872	2,072	2,281	2,521	2,771	3,005	3,246
2,156	2,381	2,612	2,871	3,132	3,369	3,604	**36**	2,077	2,287	2,505	2,756	3,016	3,259	3,509
2,370	2,605	2,846	3,116	3,389	3,636	3,882	**37**	2,285	2,503	2,729	2,989	3,257	3,509	3,766
2,575	2,817	3,064	3,341	3,621	3,874	4,126	**38**	2,481	2,704	2,936	3,201	3,476	3,732	3,994
2,752	2,996	3,246	3,526	3,808	4,064	4,318	**39**	2,649	2,875	3,109	3,376	3,652	3,909	4,173
2,884	3,132	3,385	3,668	3,953	4,211	4,468	**40**	2,776	3,004	3,240	3,509	3,786	4,044	4,309
2,983	3,236	3,494	3,783	4,074	4,338	4,600	**41**	2,865	3,097	3,338	3,612	3,896	4,159	4,429
3,062	3,323	3,590	3,889	4,190	4,463	4,734	**42**	2,935	3,175	3,423	3,706	3,998	4,270	4,548

**Table 3:** Smoothed centiles for Lithuanian birth length (cm) by sex and gestational age (GA).

Boys							GA (in weeks)	Girls						
Birth length centiles								Birth length centiles						
3	10	25	50	75	90	97		3	10	25	50	75	90	97
24.7	26.2	27.9	29.8	31.9	33.8	35.8	**22**	24.4	25.8	27.4	29.3	31.4	33.5	35.7
26.1	27.7	29.3	31.3	33.3	35.2	37.3	**23**	25.7	27.2	28.8	30.8	32.9	34.9	37.2
27.5	29.1	30.8	32.7	34.7	36.7	38.7	**24**	27.1	28.6	30.2	32.2	34.3	36.4	38.7
28.9	30.5	32.2	34.1	36.2	38.1	40.1	**25**	28.4	30.0	31.7	33.6	35.8	37.9	40.2
30.4	32.0	33.6	35.6	37.6	39.5	41.4	**26**	29.8	31.4	33.1	35.1	37.2	39.3	41.6
31.8	33.4	35.1	37.0	39.0	40.9	42.8	**27**	31.2	32.8	34.5	36.5	38.7	40.8	43.0
33.3	34.9	36.5	38.4	40.4	42.3	44.2	**28**	32.7	34.3	36.0	37.9	40.1	42.2	44.4
34.8	36.3	38.0	39.9	41.8	43.6	45.5	**29**	34.1	35.7	37.4	39.4	41.5	43.5	45.7
36.3	37.8	39.4	41.3	43.2	45.0	46.8	**30**	35.6	37.2	38.9	40.8	42.9	44.8	47.0
37.8	39.3	40.9	42.7	44.5	46.3	48.1	**31**	37.2	38.7	40.3	42.2	44.2	46.1	48.2
39.3	40.8	42.3	44.1	45.9	47.5	49.3	**32**	38.7	40.2	41.8	43.6	45.5	47.3	49.3
40.8	42.2	43.7	45.4	47.1	48.8	50.4	**33**	40.3	41.7	43.2	44.9	46.8	48.5	50.3
42.3	43.7	45.1	46.7	48.4	50.0	51.5	**34**	41.9	43.2	44.6	46.3	48.0	49.6	51.3
43.8	45.1	46.5	48.0	49.6	51.1	52.6	**35**	43.4	44.7	46.0	47.6	49.2	50.7	52.3
45.2	46.5	47.8	49.3	50.9	52.3	53.7	**36**	44.8	46.1	47.4	48.8	50.3	51.8	53.3
46.6	47.8	49.1	50.6	52.1	53.4	54.8	**37**	46.1	47.4	48.6	50.0	51.5	52.8	54.3
47.7	49.0	50.2	51.6	53.1	54.4	55.8	**38**	47.2	48.4	49.6	51.0	52.4	53.8	55.1
48.5	49.8	51.0	52.4	53.9	55.2	56.5	**39**	48.0	49.2	50.4	51.7	53.1	54.4	55.8
49.1	50.3	51.5	53.0	54.4	55.7	57.1	**40**	48.5	49.7	50.9	52.2	53.6	54.9	56.3
49.4	50.7	52.0	53.4	54.9	56.2	57.6	**41**	48.9	50.0	51.3	52.6	54.0	55.4	56.8
49.7	51.0	52.3	53.8	55.3	56.7	58.1	**42**	49.1	50.3	51.5	52.9	54.4	55.8	57.2

The 3rd, 50th and 97th Lithuanian birth weight centiles by sex and gestation were similar to those of IG-21 at extremely early gestations (24–27 weeks), especially for boys and the 97th centile (Figures 2, 3). With increasing gestation, and particularly after 37 weeks, the gap between the Lithuanian and IG-21 centiles widened to almost one centile channel or two-thirds of an SD (Figures 2, 3). The IG-21 median was close to the Lithuanian 25th centile (difference of ∼200 g), while the 97th IG-21 centile was near the Lithuanian 90th (difference of ∼350 g). At the 3rd centile, the differences among term newborns (37–40 gestational weeks) were more pronounced (200 g) than post-term (100 g) (Figures 2, 3).

For birth length, the median and lower IG-21 centiles were close to the Lithuanian centiles at early gestations, while the higher centiles differed by almost a full centile channel, i.e., 1.0–1.5 cm (Figures 4, 5). In late preterm (34–36 weeks) the differences became more pronounced and extended across all centiles, the gap widening to nearly two centile channels. The 97th IG-21 centile fell to near the Lithuanian 50th, while the 50th IG-21 centile fell to the Lithuanian 10th. At the 50th and 97th centiles, the differences at term amounted to 3 cm–4 cm, while at the 3rd centile, the difference was less at 2 cm (Figures 4, 5).

A total of 9.7% (30,859) boys were SGA based on the Lithuanian 10th centile, compared to 4.1% based on the IG-21 10th centile, less than half the prevalence (Table 4). Similarly for girls, the prevalence of SGA was 10.1% Lithuanian (30,439) vs. 4.4% IG-21. Both instruments showed the highest prevalence of SGA in very and late preterm and post-term gestations of both sexes. What is more, the older the gestational age, the larger the discrepancy between the studies. At extremely preterm gestations (24–27 weeks), IG-21 underestimated SGA in boys by up to 3.1%, and in girls up to 6.9%. The largest SGA discrepancies at term and post-term were 6.9% for boys and 8.1% for girls (Table 4). The 10th centile curves in Figures 2, 3 illustrate the discrepancies in SGA. The IG-21 10th centile is close to the Lithuanian 3rd centile until 32 weeks, and only slightly higher at later gestations (Figures 2, 3).

**Table 4:** Prevalence of small for gestational age (SGA, birth weight <10th centile) and large for gestational age (LGA, birth weight >90th centile) according to the present study's Lithuanian reference and INTERGROWTH-21st (IG-21) by sex and gestational age (GA).

Boys								GA (in weeks)	Girls							
Present study				IG-21					Present study				IG-21			
SGA		LGA		SGA		LGA			SGA		LGA		SGA		LGA	
0	0.0%	9	23.7%	0	0.0%	9	23.7%	**24**	1	2.2%	7	15.2%	1	2.2%	7	15.2%
3	3.2%	10	10.8%	1	1.1%	10	10.8%	**25**	1	1.2%	3	3.7%	0	0.0%	7	8.5%
5	3.7%	11	8.1%	3	2.2%	17	12.5%	**26**	11	7.9%	8	5.8%	7	5.0%	15	10.8%
13	6.6%	17	8.7%	7	3.6%	32	16.3%	**27**	21	12.1%	9	5.2%	9	5.2%	23	13.2%
31	10.2%	16	5.3%	10	3.3%	33	10.9%	**28**	24	10.0%	31	13.0%	14	5.9%	44	18.4%
36	12.4%	23	7.9%	19	6.5%	36	12.4%	**29**	31	12.2%	21	8.2%	15	5.9%	39	15.3%
55	12.4%	31	7.0%	23	5.2%	37	8.3%	**30**	44	10.9%	27	6.7%	22	5.4%	56	13.8%
46	9.4%	38	7.8%	18	3.7%	47	9.6%	**31**	58	13.5%	30	7.0%	36	8.4%	51	11.8%
85	10.0%	71	8.4%	35	4.1%	62	7.3%	**32**	73	10.1%	77	10.7%	28	3.9%	102	14.2%
123	11.0%	107	9.6%	42	3.8%	71	6.4%	**33**	97	10.7%	75	8.3%	49	5.4%	89	9.8%
188	10.0%	164	8.7%	102	5.4%	129	6.9%	**34**	166	10.3%	135	8.4%	112	7.0%	135	8.4%
321	11.2%	200	7.0%	199	7.0%	164	5.7%	**35**	285	11.8%	141	5.8%	233	9.6%	161	6.7%
762	13.6%	315	5.6%	443	7.9%	349	6.2%	**36**	633	12.7%	277	5.6%	496	10.0%	338	6.8%
1,293	9.8%	1,366	10.4%	653	5.0%	2,001	15.2%	**37**	1,094	9.8%	1,090	9.7%	647	5.8%	1,630	14.5%
3,195	9.4%	3,761	11.1%	1,370	4.0%	6,719	19.8%	**38**	2,946	9.9%	3,164	10.6%	1,472	4.9%	5,562	18.6%
5,928	8.7%	7,130	10.5%	2,248	3.3%	14,617	21.5%	**39**	5,976	9.3%	6,622	10.4%	2,619	4.1%	12,983	20.3%
14,231	9.8%	14,527	10.0%	5,482	3.8%	32,460	22.4%	**40**	14,561	10.3%	14,179	10.0%	5,594	3.9%	28,251	19.9%
4,085	10.0%	3,964	9.7%	1,811	4.4%	8,561	20.9%	**41**	4,026	10.3%	3,734	9.5%	1,798	4.6%	7,582	19.3%
459	18.1%	207	8.2%	283	11.2%	461	18.2%	**42**	391	17.1%	170	7.4%	207	9.1%	352	15.4%
30,859	9.7%	31,967	10.1%	12,749	4.1%	65,815	20.7%	**Total**	30,439	10.1%	29,800	9.9%	13,359	4.4%	57,427	19.1%

In contrast, the incidence of LGA was 10.1% (31,972) for boys based on the Lithuanian 90th centile as against 20.7% using the IG-21 90th centile, i.e., more than double (Table 4). Similarly in girls, LGA prevalence was 9.9% (29,810) vs. 19.1%. The differences in prevalence were particularly marked in extremely preterm (4.4%–8%) and term, post-term periods (the difference of 4.8%–12.4% more expressed for boys). The 90th centile curves in Figures 2, 3 illustrate the discrepancies in LGA between the studies. The IG-21 90th centile is near the Lithuanian 75th centile at extremely preterm and term, with a gap of almost one centile channel post-term (Figures 2, 3).

## Discussion

Growth centiles as clinical tools for assessing the infant's physical status have gained much importance in recent years. The choices between global or regional, and between customized and population-based growth references or growth standards for different populations are under debate (29, 30). The findings of our study advocate the use of regional population-based growth curves that more precisely represent the neonatal body size of Lithuanian population than the global standard IG-21 which provides the prevalence rates for SGA/LGA that differ from the true values by a factor of two in term neonates.

Our study revealed slightly better agreement in the main centiles of extremely preterm newborns (Figures 2–5). However, considering the way too modest sample size for <37 gestational weeks of the IG-21 (18), the parallels observed between the studies in the extreme centiles at early gestations should be evaluated with caution. A low sample size may explain the “waves” observed at 33 gestational weeks in the centile curves of the IG-21 (Figures 2–5).

Moreover, a larger discrepancy was observed starting from the late preterm period (34–36 gestational weeks) with certain disparities evident in mean newborn weight and length data at term (Figures 2–5). Noteworthy, our previous study comparing the Lithuanian neonatal head circumference reference with the IG-21 showed similar concerning results for term newborns (31). Likewise, other studies from different continents also found their regional neonatal reference charts to be significantly different from the neonatal standards provided by the IG-21 (24, 29, 32–35). In spite of the fact that the IG-21 found some support (36, 37), an increasing number of studies consider the IG-21 with caution including the findings of our study that support this position.

In addition, the insights drawn from the comparison of centiles between the studies when supplemented with the categorization of newborns as SGA/LGA (Table 4) demonstrate a particular relevance of the application of these standards in clinical practice. The SGA/LGA outliers were derived from the same sample of Lithuanian newborns as the regional neonatal reference curves. As shown in the results, both tails of the birth weight distribution would be strongly affected if evaluated by international standard. The possibility to evaluate the prevalence of SGA/LGA newborns at all gestational weeks may be considered a major strength of our study. While there are studies examining the newborn size categorization at term period depending on the tools used (2, 33, 38), less of them are focused on the SGA/LGA prevalence in different gestations (39). This is of a particular interest in terms of a deeper understanding of biological processes during the fetal development. Fetal growth is characterized by rapid cellular hyperplasia during the first 16 weeks, cellular hyperplasia combined with hypertrophy extending up to 32 gestational week, and cellular hypertrophy dominating afterwards till term (40). This explains the different outcomes and prognosis of being categorized as SGA at different gestational age. When the phase of cellular hyperplasia is affected (early type or symmetric hypertrophy), irreversible neurological and other sequelae of the bodily systems take place. Thus, SGA newborns born less than 32 gestational weeks experience a higher risk of death and major morbidities (41). When the phase of cellular hypertrophy is affected (late type or asymmetric hypertrophy), the processes are reversible if hypoxic ischemic encephalopathy is avoided, and the prognosis for such neonates is much more optimistic. Indeed, while newborns with birth weight of <10th centile (mild hypotrophy) may be intrinsically small because of normal biological, ethnic and other factors, it would be clinically meaningful to pay great attention to the newborns born of less than 3rd centile (severe hypotrophy) as the ones having most adverse outcomes. In this context, emphasis should be laid that adapting suitable neonatal charts is highly important for clinical practice, as failure to do so may lead to misclassification of newborns in need of timely interventions to avoid adverse consequences to their future health. Indeed, clinical decisions should be based on careful evaluation of the individual clinical case, and suitable neonatal charts are one of the tools in the decision-making algorithm.

In addition to the strengths mentioned above, our study has some limitations. Using these growth centiles for monitoring postnatal growth of preterm infants may not be realistic or ideal, because they do not reflect the initial water and weight loss that occurs in infants during the first 2–3 weeks (11, 42). Understanding this, we have set ourselves a goal of creating growth curves based on longitudinal growth data in the future.

It should also be noted that IG-21 was designed to become an international standard and included only low-risk pregnancies with women whose health and nutritional needs were met and who received adequate antenatal care in 8 socioeconomically stable developing or developed countries such as Lithuania. The data of our study included all births regardless of maternal socioeconomic status. Therefore, we believe that the main findings of our study would be even more pronounced if we had only applied the same inclusion criteria as IG-21. There are around 15% of ethnic minorities in Lithuania and Lithuanian newborns were found to be the biggest in size in comparison to newborns of other ethnic groups (43). What is more, our previous comparison (43) of newborns’ weight by mother's ethnicity in relation to education level revealed nearly no discrepancies between size of newborns from mothers with the same education level at different ethnic groups. Thus, even if ethnic differences may have influenced the discrepancies of this study, socioeconomic factors probably would be determining.

In conclusion, the global standard IG-21 should be considered with caution, as requiring validation before implementation. Instead, the findings of our study advocate the use of regional population-based neonatal centiles that more accurately represent the size of the Lithuanian newborn population. Furthermore, the prevalence of SGA/LGA at different gestations depending on the instrument used reveals the clinical importance of using local standards to benefit the most vulnerable infant populations.

## Data Availability

The data are available from the authors upon a reasonable request and with the permission of the Health Information Center of the Institute of Hygiene of Lithuania.
