# Supplementary material for: Regional references vs. international standards for assessing weight and length by gestational age in Lithuanian neonates
Source: Front Pediatr. 2023 Jun 14;11:1173685. doi: 10.3389/fped.2023.1173685 (PMC10303945; doi:10.3389/fped.2023.1173685)
Supplement: Supplementary file 1 [file Datasheet1.pdf]

Supplementary Table 1. Parameters of the LMST model (BCT distribution) for birth weight by sex and gestational age (GA).

BOYS				GA (in weeks)	GIRLS			
μ	***σ***	***υ***	***τ***		***μ***	***σ***	***υ***	***τ***
0.450	0.195	0.900	95695.480	**22**	0.422	0.216	0.597	113.139
0.575	0.189			**23**	0.539	0.209		
0.701	0.183			**24**	0.657	0.203		
0.829	0.177			**25**	0.778	0.198		
0.960	0.172			**26**	0.904	0.193		
1.098	0.168			**27**	1.038	0.188		
1.245	0.164			**28**	1.181	0.184		
1.402	0.161			**29**	1.335	0.179		
1.572	0.157			**30**	1.501	0.174		
1.756	0.154			**31**	1.680	0.169		
1.953	0.151			**32**	1.872	0.163		
2.165	0.148			**33**	2.077	0.157		
2.389	0.144			**34**	2.294	0.150		
2.625	0.139			**35**	2.521	0.144		
2.871	0.134			**36**	2.756	0.137		
3.116	0.129			**37**	2.989	0.131		
3.341	0.124			**38**	3.201	0.125		
3.526	0.118			**39**	3.376	0.119		
3.668	0.115			**40**	3.509	0.115		
3.783	0.114			**41**	3.612	0.114		
3.889	0.114			**42**	3.706	0.115		
