# Supplementary material for: Regional references vs. international standards for assessing weight and length by gestational age in Lithuanian neonates
Source: Front Pediatr. 2023 Jun 14;11:1173685. doi: 10.3389/fped.2023.1173685 (PMC10303945; doi:10.3389/fped.2023.1173685)
Supplement: Supplementary file 2 [file Datasheet2.pdf]

Supplementary Table 2. Parameters of the LMST model (BCT distribution) for birth length by sex and gestational age (GA).

BOYS				GA (in weeks)	GIRLS			
μ	***σ***	***υ***	***τ***		***μ***	***σ***	***υ***	***τ***
29.846	0.098	0.229	114.1754	**22**	29.330	0.099	-0.318	51.148
31.276	0.094			**23**	30.765	0.096		
32.706	0.090			**24**	32.199	0.093		
34.136	0.086			**25**	33.634	0.090		
35.568	0.082			**26**	35.069	0.087		
37.000	0.078			**27**	36.505	0.083		
38.432	0.075			**28**	37.941	0.080		
39.860	0.071			**29**	39.373	0.076		
41.278	0.067			**30**	40.797	0.072		
42.679	0.064			**31**	42.206	0.067		
44.057	0.060			**32**	43.592	0.062		
45.407	0.056			**33**	44.947	0.058		
46.733	0.052			**34**	46.272	0.053		
48.042	0.049			**35**	47.565	0.049		
49.339	0.045			**36**	48.821	0.045		
50.582	0.043			**37**	49.999	0.042		
51.638	0.041			**38**	50.989	0.040		
52.418	0.040			**39**	51.724	0.039		
52.965	0.040			**40**	52.234	0.039		
53.406	0.040			**41**	52.617	0.039		
53.793	0.041			**42**	52.940	0.040		
